# Timing-dependent inhibitory effects of peripheral somatosensory inputs on the primary motor cortex: A transcranial magnetic stimulation-electroencephalography study

**DOI:** 10.1016/j.ynirp.2026.100407

**Published:** 2026-09-17

**Authors:** Tomoya Kokue, Ryoki Sasaki, Kenichi Sugawara

**Affiliations:** Graduate Course of Health and Social Services, Kanagawa University of Human Services, Heisei-cho 1-10-1, Yokosuka City, Kanagawa, 238-8522, Japan

**Keywords:** Short-latency afferent inhibition, Transcranial magnetic stimulation-electroencephalography, Transcranial magnetic stimulation-evoked potential, Primary motor cortex, Electrical stimulation

## Abstract

Short-latency afferent inhibition (SAI) is a transient suppression of corticomotor excitability elicited by peripheral electrical stimulation (ES). When the interstimulus interval (ISI) between ES and transcranial magnetic stimulation (TMS) was set to 2 or 5 ms after the estimated arrival of somatosensory input in the primary somatosensory cortex, motor-evoked potentials (MEPs) were suppressed compared with single-pulse TMS. Previous studies have shown that the inhibitory activity depends on the timing between ES and TMS. However, whether subtle differences in afferent timing modulate TMS-evoked cortical responses remains unclear. To address this, we examined whether subtle ISI differences modulate cortical responses by SAI using TMS-electroencephalography (TMS-EEG). Thirty healthy adults (21.9 ± 4.4 years) participated in the current study. The ISI was determined relative to the N20 latency of somatosensory-evoked potential. MEPs were measured under three conditions: single-pulse TMS (TMS-alone), SAI with an ISI of N20 + 2 ms (SAI_N20+2_), SAI with an ISI of N20 + 5 ms (SAI_N20+5_). Subsequently, TMS-evoked potentials (TEPs) were recorded under the same three conditions and the single-pulse ES condition. MEP amplitudes were decreased in both SAI_N20+2_ and SAI_N20+5_ compared with TMS-alone (all *P* < 0.001), with greater inhibition in SAI_N20+2_ than in SAI_N20+5_ (*P* < 0.001). In contrast, the N45 component of the TEP was specifically modulated in SAI_N20+5_ compared with TMS-alone (*P* < 0.05), whereas no change was observed in SAI_N20+2_. These findings suggest that, in contrast to MEP suppression, cortical inhibition is differently modulated by subtle ISI differences, potentially reflecting distinct timing-dependent mechanisms underlying SAI.

## Graphical abstract

**Figure:**
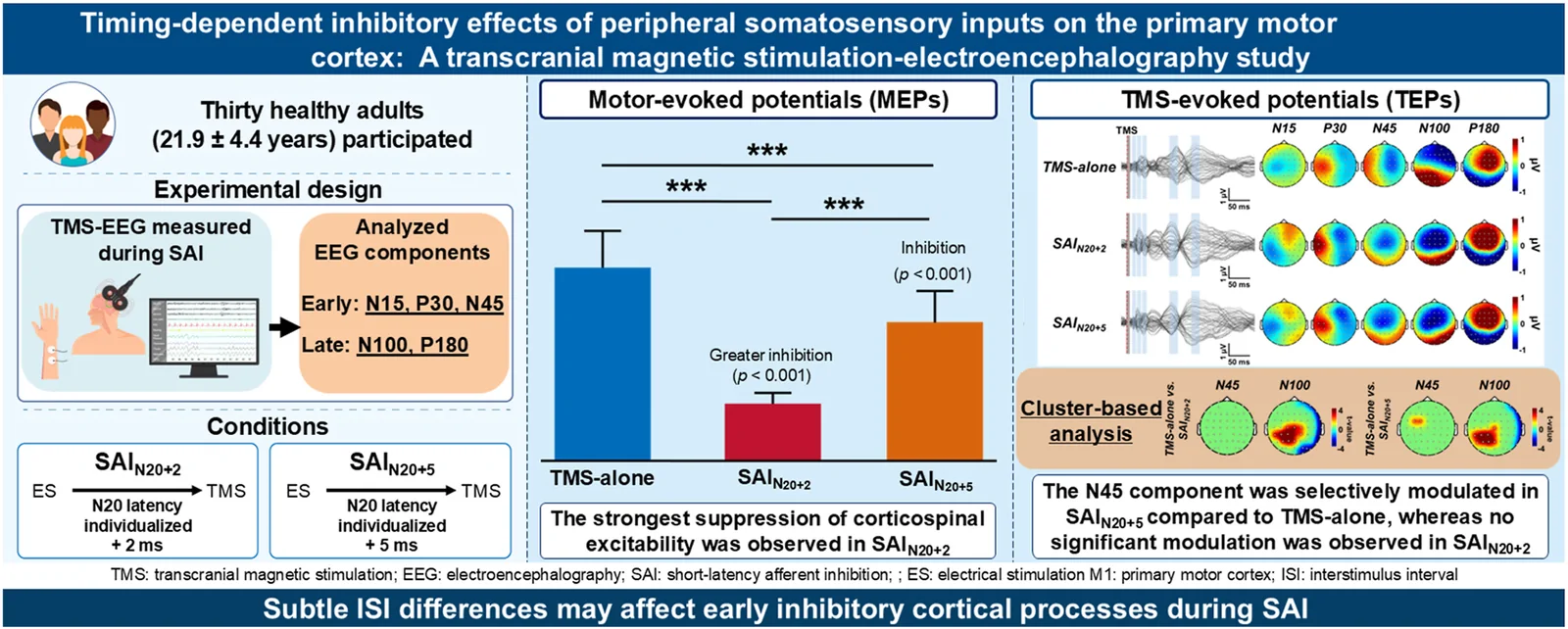

## 1 Introduction

Short-latency afferent inhibition (SAI) is a reduction in primary motor cortex (M1) excitability observed when peripheral electrical stimulation (ES) precedes transcranial magnetic stimulation (TMS) by approximately 20–28 ms (Tokimura et al., 2000). This inhibition is quantified as a reduction in MEP amplitude relative to that elicited by single-pulse MEP (Turco et al., 2018). This inhibitory response is thought to be mediated by intracortical GABAergic interneurons and the cholinergic system (Turco et al., 2018). The magnitude of inhibition varies depending on the timing between ES and TMS (Tokimura et al., 2000). In the upper limbs, corticomotor excitability is most strongly suppressed at a timing of +2 ms after the peak of the N20 component of somatosensory-evoked potentials (SEPs) recorded in response to median nerve stimulation (Tokimura et al., 2000; Stefan et al., 2002). This is because the arrival of somatosensory input to M1 coincides with the late I-wave generation by TMS (Tokimura et al., 2000). In addition, the inhibitory effect of somatosensory input on M1 excitability varies across nearby ISIs, further suggesting that SAI is a timing-dependent phenomenon sensitive to subtle differences in ES–TMS timing (Tokimura et al., 2000).

Combining TMS with electroencephalography (TMS-EEG) assesses cortical excitability and network neural dynamics across the whole brain with high temporal resolution (Tremblay et al., 2019). TMS-evoked potentials (TEPs) are EEG responses elicited by TMS over a target brain region, reflecting both local and distributed cortical activity (Cruciani et al., 2023). Several studies have used TMS-EEG to investigate cortical responses in M1 by comparing TEP components between single-pulse TMS and SAI conditions (Bikmullina et al., 2009; Ferreri et al., 2012; Noda et al., 2016). In these studies, SAI was elicited using slightly different ISIs: N20 + 5 ms with digital nerve stimulation (Bikmullina et al., 2009), and N20 + 2 ms or 3 ms with median nerve stimulation (Ferreri et al., 2012; Noda et al., 2016). Among them, only Noda et al. (2016) reported modulation of early TEP components, showing that the N45 component was increased during SAI compared with single-pulse TMS (Noda et al., 2016). This modulation may reflect increased activity of GABA_A-mediated inhibitory circuits, as suggested by pharmacological evidence (Premoli et al., 2014). Although previous studies have shown that SAI modulates cortical responses, it remains unclear whether subtle variations in the ISI are associated with changes in TMS-evoked cortical responses. MEPs primarily reflect corticomotor excitability and therefore provide limited information about intracortical inhibitory processes within M1. In contrast, TMS-EEG enables direct assessment of TMS-related cortical responses and may reveal whether differences in afferent input timing differentially modulate intracortical activity during SAI.

The current study therefore aimed to use TMS-EEG to clarify whether subtle differences in the timing of somatosensory inputs modulate cortical responses during SAI. To achieve this, we used two conditions (N20 + 2 ms [SAI_N20+2_] and N20 + 5 ms [SAI_N20+5_]) and evaluated cortical responses. We hypothesized that the N45 component would be differentially modulated in SAI_N20+2_ and SAI_N20+5_ compared with single-pulse TMS. In addition, because SAI_N20+2_ is thought to more strongly engage intracortical inhibitory circuits within M1 (Tokimura et al., 2000; Di Lazzaro et al., 2005), we further hypothesized that the N45 component would differ in magnitude and spatial distribution between SAI_N20+2_ and SAI_N20+5_. Verifying these hypotheses would clarify whether subtle differences in ISI during SAI are reflected in timing-dependent changes in cortical responses, thereby providing novel insight into the temporal mechanisms underlying sensorimotor integration.

## 2 Methods

### 2.1 Participants

Thirty healthy adults (17 males, 13 females; mean age ± SD = 21.9 ± 4.4 years) participated in this study. The Edinburgh Handedness Inventory revealed that 25 were right-handed (laterality score (mean ± standard deviation) = 86.8 ± 22.4) and 5 were left-handed (laterality score = −81 ± 23) (Oldfield, 1971). All participants had no history of neurological or psychiatric disorders and were not taking medications affecting the central nervous system. Contraindications for TMS were assessed through standard safety screening (Rossi et al., 2009). Written informed consent was obtained from all participants prior to participation. The study was conducted in accordance with the Declaration of Helsinki and approved by the Ethical Review Committee of Kanagawa University of Human Services (approval number: 18-23-45; approval date: 11 March 2025).

## 3 Experimental design

All participants completed the experiment in a single session (approximately 2 h). Participants sat in a comfortable chair inside an electrically shielded room with their hands resting on the armrests and their eyes open. During data acquisition, participants maintained the right forearm in a supinated position. The experiment began with the recording of somatosensory-evoked potentials (SEPs) elicited by ES of the right median nerve to determine the N20 peak latency. Subsequently, the individual ISI for SAI was determined based on each participant's N20 peak latency (Ferreri et al., 2012; Noda et al., 2016). For MEP measurement, three SAI conditions were measured in the following order: single-pulse TMS (TMS-alone), SAI_N20+2_, and SAI_N20+5_. This order was fixed across all participants. For TEP measurement, four conditions (TMS-alone, SAI_N20+2,_ SAI_N20+5_, and ES-alone) were assessed, and the order was pseudo-randomized within each participant. MEP and TEP recordings were performed separately because they required different TMS intensities (see the “EEG and TEP” sections for details).

### 3.1 Surface electromyography

Surface electromyographic (EMG) signals were recorded using the Neuropack X1 system (MEB-2300, Nihon Kohden, Tokyo, Japan), where they were amplified ×1000 and band-pass filtered between 20 and 1000 Hz. A notch filter (48–52 Hz) was also applied to remove line noise. Surface EMG signals were obtained from the right first dorsal interosseous (FDI) muscle using two disposable Ag/AgCl electrodes (10-mm diameter). The electrodes were positioned in a belly–tendon montage, and the ground electrode was placed over the right ulnar styloid. EMG signals were digitized at 4000 Hz using an analog-to-digital converter (PowerLab 8/30; ADInstruments, Colorado Springs, CO, USA) and stored for offline analysis.

### 3.2 TMS

Monophasic stimulation was delivered to the left M1 using a Magstim 200^2^ stimulator connected to a 9-cm figure-of-eight coil (Magstim, Dyfed, UK). The coil was positioned on the scalp at an angle of approximately 45° relative to the sagittal plane, with the handle pointing posterolaterally (Werhahn et al., 1994). The optimal stimulation site was determined as the scalp location at which TMS elicited the largest and most consistent MEPs from the resting right FDI muscle. MEPs and TEPs were recorded at 0.25 Hz. The resting motor threshold (RMT) was determined as the lowest stimulator output that produced MEPs of at least 50 μV in at least 5 of 10 consecutive trials (Rossini et al., 2015). For MEP assessment, 15 stimuli were delivered at an intensity that elicited a mean peak-to-peak MEP amplitude of 0.5–1.0 mV (MEP_1mV_). Stimulus intensity was expressed as a percentage of the maximum stimulator output.

### 3.3 SAI

ES was delivered through two surface electrodes placed over the median nerve at the wrist using a constant-current square-wave pulse of 200 μs duration. Stimulation intensity was individually adjusted to 110% of the motor threshold, defined as the minimum intensity required to evoke a visible twitch of the abductor pollicis brevis muscle following median nerve stimulation (Sugawara et al., 2024). Median nerve stimulation was used based on previous studies showing reliable elicitation of SAI in the FDI muscle (Tokimura et al., 2000; Di Lazzaro et al., 2006; Udupa et al., 2014). SAI was assessed at ISIs corresponding to N20 + 2 ms (SAI_N20+2_) and N20 + 5 ms (SAI_N20+5_), and these conditions were applied during both MEP and TEP recordings.

### 3.4 EEG and TEP

A WaveGuard EEG cap (ANT Neuro, Hengelo, Netherlands) equipped with 62 Ag/AgCl electrodes was used for EEG acquisition. The electrodes were arranged according to the international 10–10 system and connected to an EEG mylab amplifier (ANT Neuro). The CPz electrode served as the reference electrode for all recordings. Signals were online filtered (DC to 0.26 × the sampling rate), sampled at 8000 Hz, and stored for offline analysis. The impedance of all electrodes was maintained below 20 kΩ. SEPs were measured by applying 250 consecutive square wave pulses (200 μs) to the right median nerve at an interval of 333 ms. The stimulus intensity was set at 110% of the motor threshold, defined as the minimum intensity required to evoke a visible twitch of the abductor pollicis brevis muscle. TEPs were measured with 100 pulses (two blocks of 50 pulses each). TMS intensity was adjusted to 90% of each participant's RMT (Hernandez-Pavon et al., 2023). This subthreshold intensity was selected to reduce TMS-related peripheral muscle activation during EEG recordings (Biabani et al., 2019; Rocchi et al., 2021). For TMS-EEG recordings, participants wore ear defenders (Peltor Optime, 3M) with a noise reduction rating of 34 dB to reduce contamination from auditory-evoked potentials (Biabani et al., 2019; Conde et al., 2019). In addition, white noise was presented through earphones for auditory masking, and its volume was individually adjusted so that the TMS click was barely audible.

### 3.5 Data analysis

#### 3.5.1 MEP data

Each MEP trial was visually checked for muscle activity before stimulation. Trials with peak-to-peak EMG amplitudes greater than 20 μV in the 100 ms pre-TMS window were removed from the analysis because they indicated possible voluntary contraction. MEP amplitudes were assessed by peak-to-peak and reported in mV. For each condition (TMS-alone, SAI_N20+2_, and SAI_N20+5_), the mean amplitude across 15 consecutive trials was calculated.

#### 3.5.2 EEG and TEP data

EEG datasets were preprocessed and analyzed in MATLAB (R2024b; MathWorks, Natick, MA, USA) using EEGLAB (v2024.0) (Delorme and Makeig, 2004) and Brainstorm toolboxes (Tadel et al., 2011). For SEP data, channels with persistent muscle activity or external noise were removed. Subsequently, the data were band-pass filtered (1–100 Hz, 4th-order zero-phase Butterworth) and notch filtered (48–52 Hz). Next, using the Fast independent component analysis (ICA) algorithm (Hyvarinen and Oja, 2000), independent components related to blinking, eye movements, electrode noise, and muscle activity were visually identified and removed (Rogasch et al., 2017). After artifact rejection, the data were epoched from −50 to 200 ms relative to the ES (0 ms). Epochs containing muscle activity bursts or electrode noise were visually identified and excluded. Baseline correction was applied after interpolating missing electrodes and computing the average reference from 62 channels. Finally, the N20 peak latency was identified as the latency of the negative peak within 16–24 ms in the averaged SEP waveform from the selected channels (C3, CP5, CP1, P3, and CP3).

TEP data were epoched from −2000 ms to 2000 ms with the TMS trigger set at 0 ms, and baseline correction was applied to the interval from −500 ms to −30 ms. Channels exhibiting sustained high-amplitude muscle activity or noise were manually removed. Data from −2 ms to 10 ms around the TMS pulse were removed and replaced using cubic interpolation (Rogasch et al., 2017). The data were then resampled from 8000 to 1000 Hz. Epochs contaminated by muscle bursts or electrode-related noise were identified visually and excluded from further analysis. To eliminate TMS-induced artifacts, the interpolated segment from −2 ms to 10 ms was replaced with zero. An initial ICA was then performed using the Fast ICA algorithm (Hyvarinen and Oja, 2000) to remove components related to TMS-evoked muscle artifacts (Rogasch et al., 2017). A 4th-order zero-phase Butterworth filter was applied to band-pass the data between 1 and 100 Hz, and line noise was reduced using a 48–52 Hz notch filter. Source-estimate-utilizing noise-discarding algorithms (Mutanen et al., 2018) were then used to suppress remaining artifacts and reconstruct channels with missing data. A second round of ICA was then performed to automatically remove components associated with blinks, eye movements, muscle activity, or electrode noise using the TESA compselect function (Rogasch et al., 2017). Following artifact rejection, the data were separated into four conditions (TMS-alone, SAI_N20+2_, SAI_N20+5_, ES-alone). Cubic interpolation was then applied to the signal surrounding each TMS pulse. As the final preprocessing steps, the data were re-referenced to the average of all 62 channels and baseline-corrected again using the −500 to −30 ms interval.

Before the primary TEP analysis, we assessed whether ES-alone subtraction could appropriately isolate TMS-evoked cortical responses under SAI conditions (Bikmullina et al., 2009; Noda et al., 2016). ES-alone data, which were time-locked to ES onset, were temporally shifted by each participant's ISI to realign the somatosensory input relative to the TMS onset and were then subtracted from the SAI_N20+2_ and SAI_N20+5_ conditions (Bikmullina et al., 2009; Noda et al., 2016). This subtraction approach assumes that responses under the SAI conditions reflect a linear summation of TMS-evoked cortical activity and somatosensory-evoked activity. However, inspection of the ES-alone subtraction waveforms and topographies (Supplementary Fig. 1) raised concerns about this assumption, because SEP amplitudes in the ES-alone condition were larger than the TEP amplitudes observed under the SAI conditions. Therefore, ES-alone subtraction was considered unsuitable for isolating TMS-evoked cortical responses in the present study, and non-subtracted TEPs were used for the primary statistical analyses.

### 3.6 Statistical analysis

Analyses were performed using SPSS version 28 (IBM Corp., Armonk, NY, USA) or Brainstorm (Tadel et al., 2011) with the FieldTrip toolbox (Oostenveld et al., 2011). For MEP data, we compared the main effects between conditions (TMS-alone, SAI_N20+2_, SAI_N20+5_) using generalized linear mixed models (GLMMs). Data normality was first assessed using the Kolmogorov–Smirnov tests and Q–Q plots. Given the positive skew of the MEP data distribution, they were modeled using a gamma distribution with a log link function (Lo and Andrews, 2015). The model incorporated a random effect for participants. A first-order autoregressive model (AR1) was used as the covariance structure for the repeated measures. Post-hoc analyses for significant main effects were conducted using sequential Bonferroni correction with a significance threshold of 5%. MEP data are presented as estimated marginal means and 95% confidence intervals, while other data (the latency of the N20 peak and EEG data rejected by preprocessing) are presented as mean ± standard error of the mean. TEPs were analyzed using a cluster-based non-parametric permutation test with threshold-free cluster enhancement (E = 0.5, H = 2, 10,000 permutations). This method avoids the arbitrary selection of a cluster-forming threshold and enables comparisons between conditions at the spatial level (Smith and Nichols, 2009). Clusters were defined as groups of two or more adjacent electrodes among the 62 channels. To account for inter-individual variability in TEP latency, analysis windows were centered on the group-average peak latencies of each TEP component (Conde et al., 2019; Sasaki et al., 2024). Consequently, five analysis windows were selected: N15 (11–21 ms), P30 (23–33 ms), N45 (36–46 ms), N100 (99–119 ms), and P180 (151–171 ms).

As described above, ES-alone subtraction was not used in the primary analyses of the present study. To examine the potential contribution of somatosensory-evoked activity to non-subtracted TEPs under the SAI conditions, we performed Spearman's rank correlation analyses were performed between SEP amplitudes recorded in the ES-alone condition and TEP amplitudes recorded in the SAI_N20+2_ and SAI_N20+5_ conditions over the 0–200 ms post-TMS period. The resulting p-values were corrected for multiple comparisons using the false discovery rate (FDR) procedure.

## 4 Results

All 30 participants completed the experiment without adverse events. Overall, 2.5% of MEP trials were rejected due to voluntary muscle activity before TMS. The mean TMS intensities for RMT and MEP_1mV_ were 51.6 ± 6.2% and 61.3 ± 5.5%, respectively. The mean N20 peak latency was 18.4 ± 2.0 ms. During EEG preprocessing, an average of 2.1 ± 1.3 channels, 5.5 ± 4.0 (TMS-alone), 5.7 ± 2.8 (SAI_N20+2_), 5.4 ± 2.8 (SAI_N20+5_), and 6.5 ± 3.4 (ES-alone) epochs, and 2.5 ± 0.5 (first ICA) and 6.0 ± 1.3 (second ICA) independent component were removed.

### 4.1 Comparison of corticomotor excitability across conditions

**Fig. 1:**
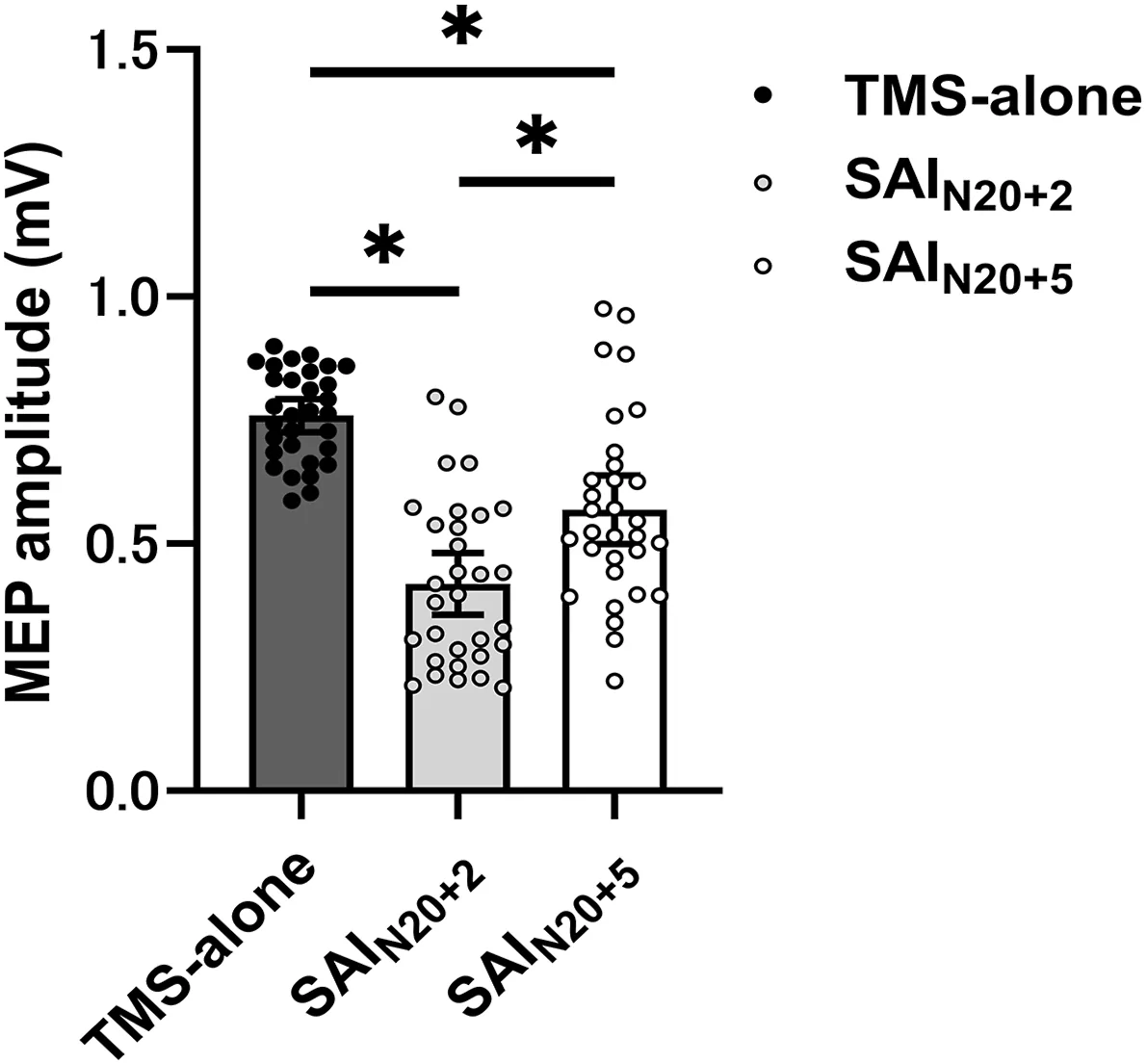
**Timing-dependent differences in corticomotor inhibition induced by SAI**. Data are presented as estimated marginal means with 95% confidence intervals, *P < 0.05. Abbreviations: MEP, motor-evoked potential; TMS-alone, only TMS was applied; SAI_N20+2_, TMS was applied at N20 + 2 ms after electrical stimulation; SAI_N20+5_, TMS was applied at N20 + 5 ms after electrical stimulation.

Fig. 1 illustrates MEP amplitudes across the three conditions. A significant main effect of condition was observed (*F*_(2,1304)_ = 30.112, *P* < 0.001). Post hoc tests revealed that MEP amplitudes were lower in both SAI_N20+2_ and SAI_N20+5_ than in TMS-alone (all *P* < 0.001). Furthermore, MEP amplitudes were lower in SAI_N20+2_ than in SAI_N20+5_ (*P* < 0.001).

### 4.2 Comparison of TEPs across conditions

**Fig. 2:**
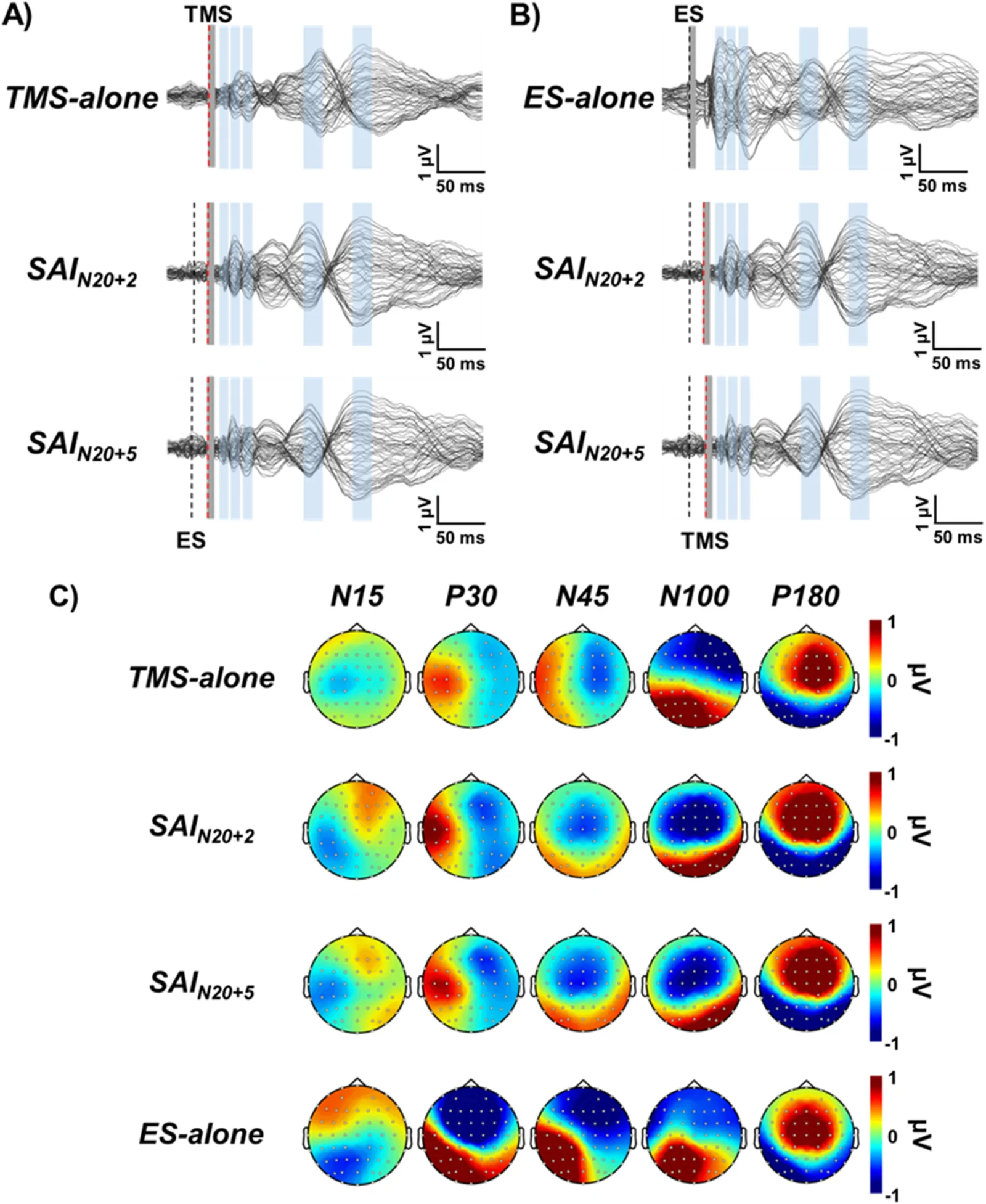
**Timing-dependent modulation of TMS-evoked cortical responses by SAI**. Grand-average TEP waveforms and TEP topographies. **(A)** TMS-locked waveforms for the TMS-alone, SAI_N20+2_, and SAI_N20+5_ conditions, with ES onset indicated in the SAI conditions. **(B)** ES-locked waveforms for the ES-alone, SAI_N20+2_, and SAI_N20+5_ conditions, with TMS onset indicated in the SAI conditions. The black and red dashed lines indicate ES and TMS onset, respectively. Waveforms show several typical components, termed N15, P30, N45, N100, and P180. The light-blue shaded ranges correspond to the time windows used in TEP analysis: N15 (11–21 ms), P30 (23–33 ms), N45 (36–46 ms), N100 (99–119 ms), and P180 (151–171 ms). (C) Topographies within the analysis intervals for the N15, P30, N45, N100, and P180 components are shown for each condition. Abbreviations: TMS-alone, only TMS was applied; SAI_N20+2_, TMS was applied at N20 + 2 ms after electrical stimulation; SAI_N20+5_, TMS was applied at N20 + 5 ms after electrical stimulation; ES-alone, only ES was applied.

**Fig. 3:**
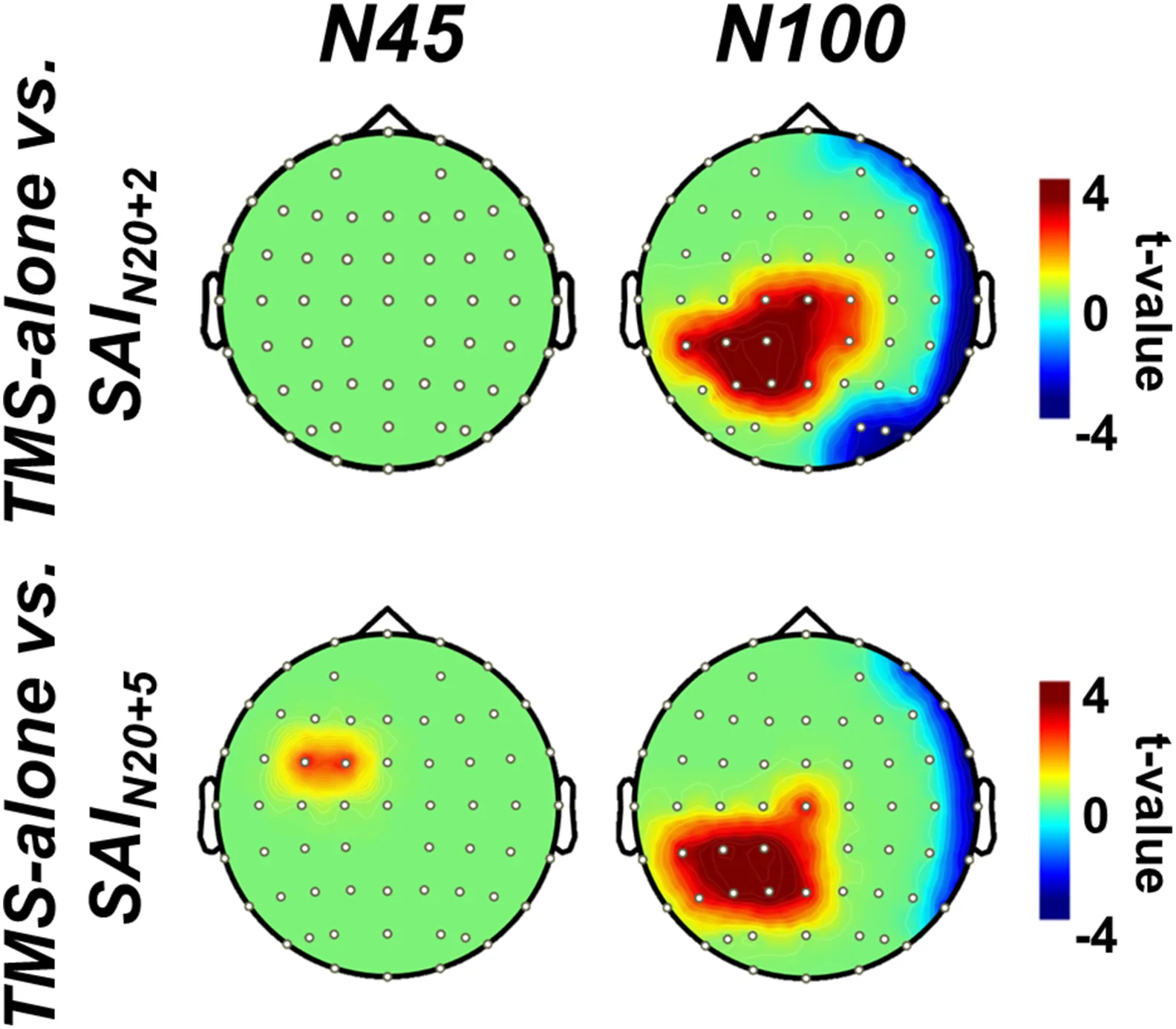
**Topographical distribution of cortical activity showing significant modulation by SAI**. A cluster-based permutation *t*-test revealed a significant positive cluster only in the N45 component for SAI_N20+5_ compared with TMS-alone. In the N100 component, both SAI_N20+2_ and SAI_N20+5_ showed significant positive and negative clusters compared with TMS-alone. No significant clusters were observed for the N15, P30, or P180 components. Abbreviations: TMS-alone, only TMS was applied; SAI_N20+2_, TMS was applied at N20 + 2 ms after electrical stimulation; SAI_N20+5_, TMS was applied at N20 + 5 ms after electrical stimulation.

Fig. 2 illustrates the grand-average data and topography of the TEP waveform elicited by TMS over M1, and Fig. 3 presents the results of the comparisons between conditions. Cluster analysis showed no significant differences between conditions in the N15, P30, or P180 components (all *P* > 0.19). In contrast, the N45 component in SAI_N20+5_ showed a significant positive cluster over the left frontal electrodes (FC1, FC3) compared with TMS-alone (Fig. 3; *P* < 0.05). No significant differences were observed in the other N45 comparisons (TMS-alone vs. SAI_N20+2_, *P* > 0.24; SAI_N20+2_ vs. SAI_N20+5_, *P* = 1). For the N100 component, both SAI_N20+2_ and SAI_N20+5_ showed significant positive clusters mainly over the central to parietal electrodes and significant negative clusters mainly over the right frontotemporal to posterior temporal electrodes compared with TMS-alone (all *P* < 0.05).

**Fig. 4:**
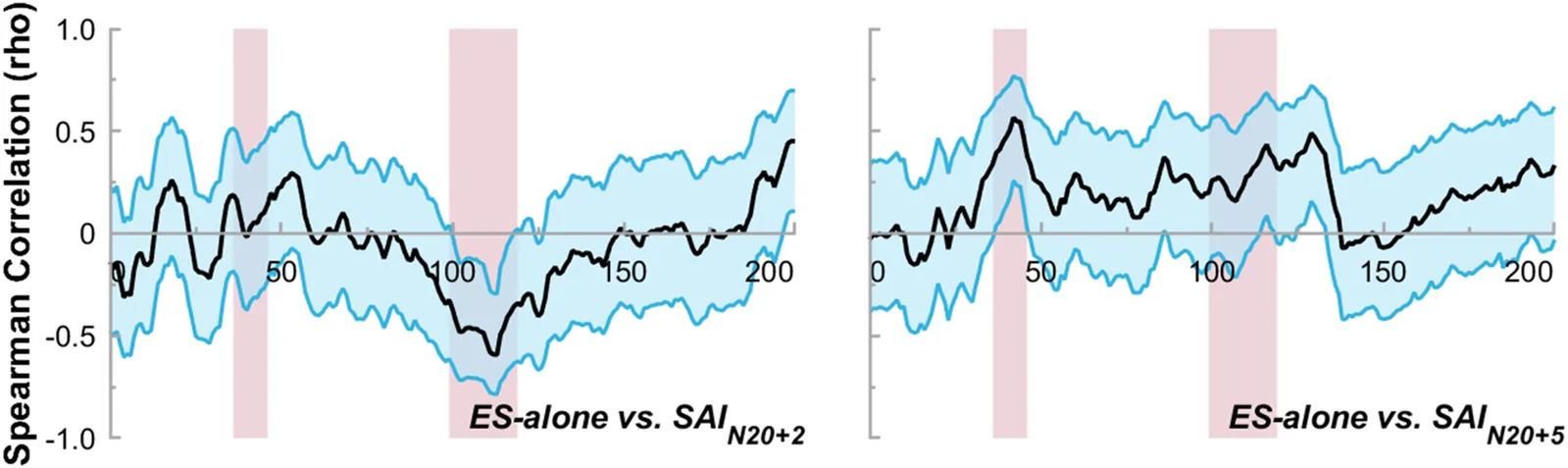
**Correlation between SEP amplitudes and TEP responses during SAI conditions.** Spearman's correlation coefficients were calculated across participants at each time point and electrode within the analyzed time window (0–200 ms). The black line indicates Spearman's rho at each time point, and the blue shaded area represents the 95% confidence interval. The shaded red area denotes the N45 (36–46 ms) and N100 (99-119 ms) analysis window. No significant correlations were observed within the analyzed time window.

Fig. 4 illustrates the results of the correlation analysis. No significant relationships were identified between SEP amplitudes and TEP amplitudes recorded in either the SAI_N20+2_ or SAI_N20+5_ (all FDR-corrected *P* > 0.05).

## 5 Discussion

This study investigated whether subtle differences in the timing of somatosensory inputs modulate TMS-evoked cortical responses elicited by SAI using TMS-EEG. MEP amplitudes were reduced in both the SAI_N20+2_ and SAI_N20+5_ conditions compared with TMS-alone, with greater inhibition in SAI_N20+2_. In contrast, TEP analysis revealed a significant modulation of the N45 component in SAI_N20+5_ compared with TMS-alone, whereas no significant change was observed in SAI_N20+2_. The N15, P30, and P180 components did not differ between conditions, whereas the N100 component was modulated in both SAI conditions compared with TMS-alone. These findings suggest that subtle differences in the timing of somatosensory inputs modulate cortical responses during SAI, as reflected by the selective modulation of the N45 component.

Modulation of cortical responses elicited by SAI has primarily been characterized by changes in the N100 component in previous TMS-EEG studies (Bikmullina et al., 2009; Ferreri et al., 2012; Noda et al., 2016). For example, Bikmullina et al. (2009) reported that attenuation of MEP amplitudes during SAI was significantly correlated with a reduction in N100 amplitude (Bikmullina et al., 2009). Similarly, Ferreri et al. (2012) showed that SAI condition attenuated the N100 component, particularly over fronto-central electrodes (Ferreri et al., 2012). The N100 component has been linked to GABA_B receptor–mediated intracortical inhibition (Premoli et al., 2014), and its attenuation during SAI is thought to reflect modulation of GABA_B-dependent inhibitory mechanisms (Bikmullina et al., 2009; Ferreri et al., 2012). Consistent with these previous studies, the present study also observed N100 modulation in both SAI conditions compared with TMS-alone. In contrast, evidence regarding earlier TEP components during SAI remains limited. Among previous TMS-EEG studies, Noda et al. reported significant modulation of early TEP components during SAI (Noda et al., 2016). They showed that in the M1-SAI condition with an ISI of N20 + 2 ms, significant increases in both the N45 and N100 components were observed, whereas the P30 component remained unchanged (Noda et al., 2016). In the present study, modulation of the N45 component was observed in the SAI_N20+5_ condition. Given that pharmacological studies have shown that the N45 component is sensitive to modulation of GABA_A receptors (Premoli et al., 2014), this finding suggests that SAI may influence not only later N100-related inhibitory mechanisms but also earlier cortical inhibitory processes reflected in the N45 component.

A related methodological concern is that activity within the N45 time window may be affected by TMS-related peripheral muscle activation. A TMS-EEG source-localization study using suprathreshold M1 stimulation suggested that the N45 time window may include activity associated with TMS-related peripheral muscle activation (Ahn and Frohlich, 2021). This issue is important to consider; however, the present study was designed to minimize such contamination by using subthreshold TMS at 90% of the resting motor threshold for EEG recordings (Biabani et al., 2019; Rocchi et al., 2021), in contrast to the suprathreshold stimulation used in that study. Therefore, the use of subthreshold TMS makes it unlikely that this factor primarily accounts for the N45 modulation observed in the present study. Together, these findings suggest that cortical modulation during SAI is not limited to the later N100 component but may also involve early cortical responses reflected in the N45 component, depending on the timing of afferent input. In contrast to Noda et al. (2016), the present study did not observe significant modulation of the N45 component in the SAI_N20+2_ condition. Although the present findings diverged from those of Noda et al. (2016), key methodological elements were determined based on their study. In particular, the ISI was defined individually based on the latency of the N20 peak obtained from SEP recordings. Accordingly, differences in ISI determination are unlikely to explain the absence of N45 modulation in the SAI_N20+2_. In addition, the present study included a larger sample (n = 30) than Noda et al. (2016) (n = 12), suggesting that insufficient statistical power is less likely to explain the absence of N45 modulation. However, methodological differences also exist between the studies. One important difference is the TMS intensity to record TEPs. Noda et al. (2016) applied suprathreshold stimulation sufficient to elicit MEP amplitudes of approximately 1 mV (Noda et al., 2016), whereas the present study used subthreshold stimulation (90% RMT). Given that TMS-evoked EEG responses increase nonlinearly with stimulation intensity (Komssi et al., 2004), the suprathreshold stimulation used by Noda et al. (2016) may have produced larger early TEP responses, making SAI-related modulation of the N45 component more readily detectable than in the present study. However, intensities exceeding 100% RMT increase the risk of confounding signals, including peripheral somatosensory responses (Biabani et al., 2019; Conde et al., 2019; Beck et al., 2024). Thus, TEP amplitude and artifact contamination represent an inherent trade-off. This factor may have contributed to differences between the studies, and future studies are needed to clarify its influence on cortical responses.

Previous TMS studies have shown that the inhibitory effect of somatosensory input on M1 excitability varies depending on the ISI between ES and TMS (Tokimura et al., 2000; Stefan et al., 2002). In the present study, modulation of the N45 component was observed specifically in the SAI_N20+5_, but not in the SAI_N20+2_. This finding suggests that the additional 3-ms delay between afferent input and TMS was associated with changes in early TMS-evoked cortical responses during SAI. One mechanism suggested by the present pattern of results is a timing-dependent interaction between somatosensory afferent input and local intracortical inhibitory circuits. This interpretation is consistent with TMS studies showing that intracortical inhibition depends on the interval between conditioning and test inputs, reflecting the time-dependent recruitment of distinct inhibitory cortical processes (Ziemann et al., 2015). In the SAI paradigm, somatosensory afferent input is thought to interact with TMS-induced activity in M1, including late I-wave–generating interneuron circuits (Tokimura et al., 2000). Therefore, even a small shift in ES–TMS timing may alter the temporal overlap between afferent input and TMS-evoked local inhibitory activity. Given that the N45 component is sensitive to modulation of GABA_A receptors (Premoli et al., 2014), the selective N45 modulation observed in the SAI_N20+5_ condition may reflect a timing-dependent interaction between afferent input and GABA_A-related inhibitory processes. In addition, previous paired associative stimulation (PAS) studies suggest that at ES–TMS intervals of 25 ms or longer, somatosensory input may influence M1 through pathways that are partly distinct from the S1-mediated pathway (Dubbioso et al., 2015; Hamada et al., 2012). For example, Hamada et al. (2012) showed that cerebellar anodal transcranial direct current stimulation differentially affected paired associative stimulation (PAS) -induced changes in corticomotor excitability depending on whether the ES–TMS interval was 21.5 or 25 ms (Hamada et al., 2012). Dubbioso et al. (2015) further showed that PAS with an ES–TMS interval of 25 ms increased corticomotor excitability in healthy adults, whereas this effect was absent in patients with cerebellar degeneration (Dubbioso et al., 2015). These findings suggest that a cerebellar-related pathway may contribute to the selective N45 modulation observed in the SAI_N20+5_ condition. However, future studies combining SAI TMS-EEG with cerebellar interventions are needed to determine whether cerebellar-related pathways contribute to the selective N45 modulation observed in the SAI_N20+5_ condition.

Importantly, these timing-dependent effects were expressed differently in corticomotor excitability and in the N45 component. The strongest suppression of corticospinal excitability was observed in the SAI_N20+2_, consistent with previous studies demonstrating robust inhibition at this interval (Tokimura et al., 2000; Stefan et al., 2002). Tokimura et al. proposed that this suppression may involve inhibition of late I-wave–generating interneuron circuits in M1 (Tokimura et al., 2000). In contrast, only the SAI_N20+5_ induced modulation of the N45 component. These findings suggest that corticospinal output and intracortical responses, while both influenced by afferent timing, reflect partially distinct inhibitory processes. While MEPs reflect the activation of corticospinal neurons following transsynaptic excitation of pyramidal neurons in M1, with action potentials propagating along the corticospinal tract to the target muscles (Di Lazzaro et al., 2008), TEPs primarily reflect excitatory and inhibitory postsynaptic potentials within cortical circuits (Hernandez-Pavon et al., 2023; Di Lazzaro et al., 2008). The dissociation observed here suggests that, although corticospinal excitability reflects timing-dependent modulation, it may not fully capture the distinct intracortical inhibitory processes revealed by TEPs, highlighting the additional insight provided by TMS-EEG.

One important limitation of the present study concerns the interpretation of TEP data. First, statistical analyses were performed using non-subtracted TEP data because ES-alone subtraction was considered unsuitable in the present study. Specifically, the ES-alone SEP amplitudes were larger than the TEP amplitudes observed under the SAI conditions, suggesting that the SAI waveforms could not be explained as a simple linear summation of TMS-evoked cortical activity and somatosensory-evoked activity. Therefore, subtracting the ES-alone waveform may have distorted, rather than isolated, TMS-evoked cortical responses. This issue needs to be considered in light of the methodological context of previous TMS-EEG studies using the SAI paradigm (Bikmullina et al., 2009; Ferreri et al., 2012; Noda et al., 2016). Previous studies were mainly conducted using suprathreshold TMS (Bikmullina et al., 2009; Ferreri et al., 2012; Noda et al., 2016), whereas more recent methodological studies have suggested that subthreshold TMS is associated with reduced sensory-related contributions to TMS-evoked cortical responses compared with suprathreshold TMS (Biabani et al., 2019; Rocchi et al., 2021). In the present study, subthreshold TMS was therefore used for TEP recordings to reduce TMS-related peripheral muscle activation. However, to our knowledge, no previous study had examined SAI-related TEP modulation using subthreshold TMS. Therefore, under the subthreshold TMS protocol, it was difficult to determine a priori whether subtraction would be appropriate, because the amplitude changes of SEP and TEP components were not known in advance. To further address this issue, we performed correlation analyses between SEP amplitudes in the ES-alone and TEP amplitudes in the SAI_N20+2_ and SAI_N20+5_. These analyses showed no significant correlations, suggesting that the early TEP responses during the SAI conditions were not simply explained by individual differences in SEP amplitude. Nevertheless, the absence of significant correlations does not rule out the possibility that SEP-related activity contributed to the early TEP components. Therefore, in the absence of an appropriate ES-alone subtraction or equivalent sensory control condition, the early TEP responses observed during the SAI conditions cannot be unequivocally attributed to TMS-evoked cortical activity. Therefore, the early TEP responses observed during the SAI conditions may not reflect purely TMS-evoked cortical activity. Future studies are needed to include additional control conditions, such as an ISI of N20 + 10 ms, as used by Ferreri et al. (2012), in which SEP-related activity elicited by peripheral somatosensory input is still present, but SAI is less likely to occur. Such a condition would help to better dissociate cortical modulation during SAI from SEP-related activity. Second, TEPs were measured using subthreshold TMS at 90% RMT. Although this approach was selected to reduce TMS-related peripheral muscle activation during EEG recording, the effects of SAI on TEPs may differ between subthreshold and suprathreshold TMS. Therefore, the present findings obtained with subthreshold TMS cannot be directly generalized to SAI responses elicited by suprathreshold TMS. Finally, MEP measurements were performed in a fixed rather than randomized order. Because the SAI_N20+2_ condition was always measured before the SAI_N20+5_ condition, order-related effects, such as time-dependent changes in corticomotor excitability, cannot be completely excluded. Nevertheless, the observed effect, with stronger suppression in SAI_N20+2_ than in SAI_N20+5_, was consistent with previous SAI studies (Tokimura et al., 2000; Stefan et al., 2002). Thus, order effects are unlikely to fully account for the observed ISI-dependent MEP suppression.

In conclusion, this study showed that subtle differences in afferent timing are associated with changes in early TEP responses during SAI, as reflected by modulation of the N45 component. These findings suggest that subtle variations in the ISI between ES and TMS may be associated with early cortical responses during SAI, potentially reflecting timing-dependent interactions between afferent input and local cortical circuits involved in MEP suppression.

## CRediT authorship contribution statement

**Tomoya Kokue:** Writing – original draft, Visualization, Project administration, Methodology, Investigation, Formal analysis, Data curation. **Ryoki Sasaki:** Writing – original draft, Supervision, Project administration, Methodology, Investigation, Funding acquisition, Formal analysis, Data curation, Conceptualization. **Kenichi Sugawara:** Writing – review & editing, Supervision, Methodology, Investigation, Data curation.

## Funding sources

This work was supported by the 10.13039/501100001691JSPS KAKENHI [20K11287 to K.S. and 24K20444 to R.S.]. R.S. was supported by a JSPS Research Fellowship for Young Scientists (222270200002MY).

## Declaration of competing interest

The authors declare that they have no known competing financial interests or personal relationships that could have appeared to influence the work reported in this paper.

## Data Availability

The authors do not have permission to share data.
