# Supplementary material for: Timing-dependent inhibitory effects of peripheral somatosensory inputs on the primary motor cortex: A transcranial magnetic stimulation-electroencephalography study
Source: Neuroimage Rep. 2026 Sep 17;6(4):100407. doi: 10.1016/j.ynirp.2026.100407 (PMC13602058; doi:10.1016/j.ynirp.2026.100407)
Supplement: Supplementary file 1 [file mmc1.docx]

**Supplementary Material**

**Supplementary Fig. 1: Timing-dependent modulation of TMS-evoked cortical responses by peripheral somatosensory input after subtraction of SEP.** Butterfly plots of grand-average TEP waveforms after SEP subtraction in the SAI conditions. Abbreviations: SAI_N20+2_, TMS was applied at N20+2 ms after electrical stimulation; SAI_N20+5_, TMS was applied at N20+5 ms after electrical stimulation.
